# STED: flexible cross-modal topic modeling infers cell-type-specific regulatory landscapes from bulk epigenomics

**DOI:** 10.1093/bib/bbag347

**Published:** 2026-06-30

**Authors:** Yunxi Liao, Dongyu Zhao

**Affiliations:** Department of Biomedical Informatics, School of Basic Medical Sciences, Peking University, 38 Xueyuan Road, 100191 Beijing, China; State Key Laboratory of Vascular Homeostasis and Remodeling, Peking University, 38 Xueyuan Road, 100191 Beijing, China; Department of Biomedical Informatics, School of Basic Medical Sciences, Peking University, 38 Xueyuan Road, 100191 Beijing, China; State Key Laboratory of Vascular Homeostasis and Remodeling, Peking University, 38 Xueyuan Road, 100191 Beijing, China

**Keywords:** topic modeling, cross-modal prediction, epigenomics, deconvolution, cell-type-specific signals

## Abstract

Deciphering cell-type-specific epigenetic landscapes within heterogeneous tissues is restricted by the sparsity, high cost, and limited throughput of current single-cell epigenomic technologies. To bridge the gap between massive legacy bulk epigenomic data and cellular resolution, we present STED (Single-cell Topic modeling and Epigenetic deconvolution), a flexible computational framework that reconstructs cell-type-specific regulatory signals by leveraging single-cell transcriptomic references. Methodologically, STED introduces a versatile topic modeling architecture: users can employ the information-theoretic correlation explanation algorithm to robustly mitigate transcriptomic sparsity, or innovatively adapt BERTopic—a large language model-based framework—to capture high-dimensional semantic cellular states. STED couples these latent regulatory topics with a physics-based “gene activity score” transformation, which acts as a cross-modal bridge to deconvolve bulk chromatin accessibility (ATAC-seq) and histone modification (ChIP-seq/CUT&Tag) profiles. Extensive benchmarking across diverse datasets—including human peripheral blood mononuclear cells (PBMCs), mouse brain, and zebrafish inner ear—demonstrates that STED significantly outperforms existing rigid-reference tools in accuracy and robustness against cross-platform batch effects. In a hematopoietic stem cell differentiation model, STED successfully identifies cell-type-specific transcription factor binding motifs and differential peaks associated with lineage commitment. We further demonstrate STED’s biological utility by uncovering tumor-specific super-enhancers in colorectal cancer—a mechanism obscured in bulk signals. STED thus provides a scalable, foundation-model-empowered solution for dissecting gene regulatory networks in complex tissues.

## Introduction

 Epigenetic mechanisms orchestrate cellular identity and gene expression, and their dysregulation is a hallmark of diverse pathologies ranging from cancer to neurodegenerative disorders [1–3]. However, elucidating these mechanisms within complex tissues is challenging. Traditional bulk assays (e.g. ChIP-seq and ATAC-seq), while providing sequencing depth, inherently average signals across diverse cell populations. This obscures cell-type-specific heterogeneity, as dynamic shifts in cell abundance can mimic or mask true regulatory alterations [4, 5].

While single-cell RNA sequencing (scRNA-seq) has revolutionized the resolution of cellular composition [6], translating this resolution to the epigenome remains a significant hurdle. Emerging single-cell epigenomic technologies (e.g. scATAC-seq) hold transformative potential but are currently hindered by extreme data sparsity (high dropout rates), limited throughput, and prohibitive costs, rendering them difficult to apply to large clinical cohorts [7, 8]. Conversely, the scientific community possesses vast repositories of legacy bulk epigenomic datasets that remain underutilized. Although deconvolution methods for bulk ATAC-seq have recently been proposed (e.g. EPI-ATAC [9] and DeconPeaker [10]), they exhibit significant limitations in flexibility. These approaches typically rely on rigid, predefined reference peaks derived from specific tissues (e.g. PBMCs), restricting their generalizability to other biological contexts.

To bridge this gap, integrating scRNA-seq references with bulk epigenomic profiling emerges as a strategic solution. Grounded in the premise that tissues share conserved cellular programs across modalities, we developed STED (Single-cell Topic modeling and Epigenetic deconvolution), a modular framework for transcriptome-to-epigenome transfer. The key idea in STED is not the introduction of a single new topic model, but the construction of a shared latent interface that connects three tasks within one workflow: (i) learning biologically interpretable topic representations from scRNA-seq references; (ii) projecting bulk epigenomic signals into the same topic space through a GAS bridge; and (iii) using this shared representation to estimate cell-type proportions and reconstruct cell-type-specific activating regulatory signals. Under this design, CorEx, latent Dirichlet allocation (LDA), and BERTopic function as alternative scTopic backends whose outputs are standardized before downstream deconvolution. By separating backend-specific topic discovery from a common cross-modal transfer procedure, STED provides a scalable and flexible solution for resolving epigenetic heterogeneity in both healthy and disease-associated tissues.

## Materials and methods

### The single-cell topic modeling and epigenetic deconvolution workflow

**Figure 1:**
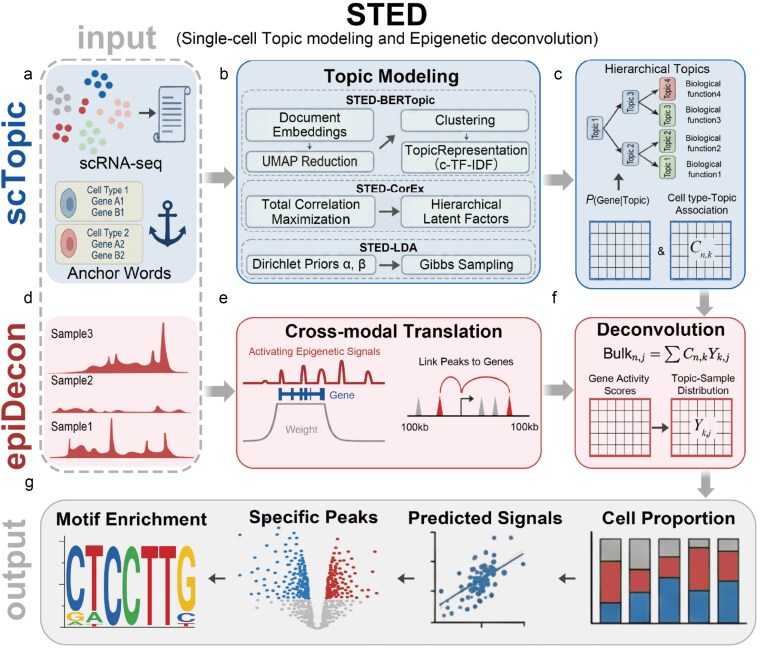
Overview of the STED framework. The STED workflow integrates single-cell transcriptomic programs with bulk epigenomic deconvolution through two primary modules: scTopic (panels A–C) and epiDecon (panels D–F). (a) Input Processing: scRNA-seq data are processed using an anchor word strategy, conceptualizing cells as documents and genes as words. (b) Topic Modeling (scTopic): The framework implements three interchangeable backends (CorEx, LDA, and BERTopic) to discover latent gene modules. Despite distinct mathematical foundations, their outputs are standardized via a backend abstraction layer. (c) Hierarchical Structure and Unified Output: This panel illustrates the conceptual hierarchical topic trees (derived intrinsically for CorEx, or via *post-hoc* clustering for LDA/BERTopic). The abstraction layer generates a standardized cell type–topic association matrix ($C_{n,k}$), anchoring latent topics to biological cell types. (d–f) Cross-modal Translation and Deconvolution (epiDecon): The epiDecon module accepts bulk epigenomic profiles (d) and translates them into a pseudo-transcriptomic space by calculating GAS (e). Utilizing the latent organization derived from scRNA-seq, cell-type proportions, and topic–sample distributions ($Y_{k,j}$) are inferred via the formulation $\mathrm{Bulk}_{n,j} = \sum C_{n,k}Y_{k,j}$ (f). (g) Downstream Applications: The final outputs include motif enrichment analysis, identification of cell-type-specific peaks, predicted signal correlation, and estimated cell-type proportions.

STED is a probabilistic framework enabling cell-type deconvolution and signal inference from bulk epigenomics using scRNA-seq references. The workflow consists of two synergistic modules (Fig. 1):

**1. scTopic (latent structure learning)**: This module extracts cell-type-specific transcriptional signatures by treating single cells as “documents” and genes as “words” (Fig. 1a). It offers a versatile modeling framework that supports both probabilistic approaches (e.g. CorEx) and foundation-model-based strategies (e.g. BERTopic). By learning latent distributions, scTopic derives robust, denoised representations of cellular identity, resolving hierarchical relationships among cell states (Fig. 1b).

**2. epiDecon (cross-modal deconvolution)**: This module performs cross-modal translation. First, it transforms bulk epigenomic signals into a “pseudo-transcriptomic” space using Gene Activity Scores (GAS), integrating chromatin accessibility with distance-weighted regularization. Second, utilizing cell type–topic priors learned by scTopic, epiDecon applies Bayesian inference to estimate cell-type proportions. Finally, it reconstructs cell-type-specific epigenetic landscapes to identify active regulatory elements (Fig. 1c and d).

### scTopic: topic modeling of single-cell RNA sequencing data

#### A unified abstraction across topic-model backends

The three topic-learning backends used in STED do not share the same generative assumptions. Correlation explanation (CorEx) optimizes information-theoretic dependence, LDA uses a Dirichlet-multinomial topic model, and BERTopic combines embedding-based clustering with c-TF-IDF weighting. Accordingly, we do not assume that their internal latent spaces are identical. Instead, STED standardizes their outputs into a common abstraction that can be passed to epiDecon.

For a single-cell reference with $N$ cell types, $K$ topics, $G$ genes, and $M$ cells, each backend produces: (i) a gene–topic matrix $W^{(g)} \in \mathbb{R}^{G \times K}$ describing topic-associated genes; (ii) a cell–topic matrix $\Theta ^{(sc)} \in \mathbb{R}^{K \times M}$ describing topic usage per cell; and (iii) a topic–cell-type matrix $C \in \mathbb{R}^{N \times K}$, where 


(1) \begin{align*}& C_{n,k} = P(\mathrm{cell\ type}_{n} \mid \mathrm{topic}_{k}).\end{align*}

This matrix is estimated by aggregating topic assignments across annotated cells in the scRNA-seq reference. In practice, $C$ is the quantity used to connect topic representations with biological cell types during deconvolution.

Under this abstraction, the practical role of each backend is to provide a different route for estimating the same transferable objects. CorEx is used when robustness to sparse count structure is prioritized, LDA provides a classical probabilistic baseline, and BERTopic provides an embedding-based alternative that can exploit semantic structure captured by single-cell foundation models. The downstream epiDecon module uses the standardized outputs $(W^{(g)}, \Theta ^{(sc)}, C)$ and is, therefore, not restricted to a single topic-learning algorithm. In practice, backend consistency is evaluated by comparing downstream deconvolution accuracy [Pearson correlation coefficient (PCC) and Root Mean Square Error (RMSE)] and biological coherence [cell-type marker enrichment in top-topic genes, measured by normalized mutual information (NMI) against annotated labels] across backends applied to the same dataset.

#### Correlation explanation

Let $X_{G}=\{X_{g}: g \in G\}$ denote the expression variables for a selected gene set $G$. CorEx [11] discovers biologically meaningful topics by maximizing the Total Correlation (TC), an information-theoretic metric quantifying dependence among genes. Formally, TC is defined as the Kullback–Leibler divergence between the joint distribution and the product of marginals: 


(2) \begin{align*}& \mathrm{TC}(X_{G}) = \sum_{i \in G} H(X_{i}) - H(X_{G}) = D_{\mathrm{KL}} \left( p(x_{G}) \parallel \prod_{i \in G} p(x_{i}) \right)\end{align*}

where $H(\cdot )$ denotes entropy. The objective is to identify latent topic variables $Z_{1},\ldots ,Z_{K}$ that maximally explain the dependencies within gene groups $G_{j}$. The optimization problem is written as: 


(3) \begin{align*}& \max_{\substack{G_{j}, \\ p(z_{j} \mid x_{G_{j}})}} \sum_{j=1}^{K} \mathrm{TC}(x_{G_{j}}; Z_{j})\end{align*}

To solve this efficiently, STED employs a soft assignment strategy in which $\rho _{g,k}$ denotes the degree to which gene $g$ is associated with topic $k$. The iterative update maximizes the mutual information $I(X_{g}: Z_{k})$ between genes and topics: 


(4) \begin{align*}& \rho_{g,k}^{(t)} = \exp \left( \lambda^{(t)} \left( I(X_{g}: Z_{k}) - \max_{\bar{k}} I(X_{g}: Z_{\bar{k}}) \right) \right)\end{align*}

where $\lambda ^{(t)}$ controls the sharpness of the soft-max function. We use $\rho $ here to distinguish the CorEx gene–topic assignment parameter from the Dirichlet hyperparameters used in LDA below.

STED further enhances CorEx with two mechanisms:

1. **Anchor-word guidance:** predefined marker genes are used as soft priors to bias selected topics toward known cell identities while preserving unsupervised structure discovery.
2. **Hierarchical modeling:** By recursively treating topic distributions of one layer as inputs for the next, we maximize explained TC across layers ($\sum \mathrm{TC}(Y; Z_{l})$), yielding an intrinsic hierarchy from concrete functions to higher-order abstractions.

#### Latent Dirichlet allocation

Alternatively, STED incorporates LDA [12] via collapsed Gibbs sampling. Let $d$ index cells, $g$ index genes, and $k$ index topics. The topic assignment for the $i$th observed gene token in cell $d$ is approximated by: 


(5) \begin{align*}& P(T_{d,i}=k \mid \mathbf{T}^{-d,i}, \alpha, \beta) \propto \frac{n_{k,g}^{-d,i} + \beta_{g}}{n_{k}^{-d,i} + \sum_{g} \beta_{g}} \times \frac{n_{d,k}^{-d,i} + \alpha_{k}}{n_{d}^{-d,i} + \sum_{k^{\prime}=1}^{K} \alpha_{k^{\prime}}}\end{align*}

where $\alpha _{k}$ is the Dirichlet prior over topic usage in cells, $\beta _{g}$ is the Dirichlet prior over genes within topics, and $n_{(\cdot )}^{-d,i}$ denotes counts excluding the current token.

To integrate biological priors, we implement Anchored Topic Modeling. We introduce a guidance parameter $L_{k,v}$ to constrain updates for anchor genes. Specifically, for an anchor gene $v$ associated with target topic $k^{\prime}$, we assign a rigid weight $\lambda $: 


(6) \begin{align*}& L_{k,v} = \begin{cases} \lambda, & \mathrm{if}\ k = k^{\prime} \\ 0, & \mathrm{otherwise} \end{cases}\end{align*}

This parameter probabilistically biases anchor genes toward their designated topics. Because standard LDA is a flat topic model, hierarchical relations are not intrinsic; in STED, they are obtained by *post-hoc* clustering of the inferred topic representations.

#### Model selection and evaluation

Both CorEx and LDA are sensitive to initialization and the hyperparameter $K$ (number of topics). We optimize $K$ and select the best model run using backend-appropriate metrics: TC for CorEx and UMass coherence for LDA. The pairwise UMass coherence between genes $g_{i}$ and $g_{j}$ is defined as: 


(7) \begin{align*}& C_{\mathrm{UMass}}(g_{i}, g_{j}) = \log \frac{DF(g_{i}, g_{j}) + \epsilon}{DF(g_{j}) + \epsilon}\end{align*}

where $DF(g_{i}, g_{j})$ denotes the co-occurrence count. The overall coherence for topic $t$ is calculated as the average pairwise score: $\mathrm{score}_{\mathrm{UMass}}(t) = \frac{2}{m(m-1)} \sum _{i<j} C_{\mathrm{UMass}}(g_{i}, g_{j})$.

To validate biological relevance, we quantify the agreement between topic-derived cell clusters $A$ and ground-truth cell-type labels $B$ using NMI. Here, “cluster” refers to groups of cells partitioned in the learned topic representation space, either directly by the backend or by downstream clustering of cell–topic representations: 


(8) \begin{align*}& \mathrm{NMI}(A, B) = \frac{2 I(A; B)}{H(A) + H(B)}\end{align*}

where $I(A; B)$ denotes mutual information and $H(\cdot )$ represents entropy. NMI values range from 0 to 1, with higher values indicating better alignment with biological annotations.

#### Foundation-model-based topic modeling (BERTopic)

To leverage semantic context beyond probabilistic counts, we integrated a BERTopic module:

1. **Embeddings:** High-dimensional cell embeddings are generated using single-cell foundation models (e.g. scFoundation, scBERT).
2. **Clustering:** Embeddings are reduced via UMAP and clustered using HDBSCAN or Leiden to define topics.
3. **Inverse document frequency (c-TF-IDF):** in c-TF-IDF, each topic is treated as a pseudo-document formed by cells within a cluster. Gene weights $g$ are then computed within each topic $c$ (rather than globally) to highlight topic-specific genes:
  (9) \begin{align*}& \begin{aligned} \mathrm{c-TF-IDF}(g, c) &= \mathrm{TF}(g, c) \times \log \left( 1 + \frac{K}{\mathrm{DF}(g)} \right) \\ \mathrm{TF}(g, c) &= \frac{\mathrm{Count}(g, c)}{\sum_{g^{\prime} \in c} \mathrm{Count}(g^{\prime}, c)} \end{aligned}\end{align*}
  where $\mathrm{Count}(g, c)$ denotes the total number of occurrences of gene $g$ in topic cluster $c$, $K$ is the total number of topics, and $\mathrm{DF}(g)$ represents the number of topics that contain gene $g$.

For BERTopic, hierarchical relations are not learned by a generative model. Instead, they are derived by clustering topic representations such as c-TF-IDF profiles or topic embeddings. Therefore, across STED backends, the hierarchy is a biologically organized relationship among topics rather than a single model-specific latent tree.

#### Biological interpretation of transcriptional topics

To interpret the inferred topics, we first identified “core genes” by applying $k$-means clustering to the gene–topic weight vectors $P(\mathrm{gene} \mid \mathrm{topic})$ and retaining the high-weight cluster. Functional enrichment analysis was performed on these core genes using Gene Ontology Biological Processes retrieved via the *msigdbr* package. Significance was assessed using empirical permutation tests with $P$-values corrected for multiple testing (Benjamini–Hochberg adjusted $P <.05$). Alternatively, STED supports Gene Set Enrichment Analysis (GSEA) using the *clusterProfiler* package. Finally, to link topics to specific cellular identities, we performed hierarchical clustering on the cell type–topic posterior probability matrix $C_{n,k} = P(\text{cell type}_{n} \mid \mathrm{topic}_{k})$.

### epiDecon: cross-modal deconvolution and signal inference

#### Cross-modal transformation via gene activity scores

To align epigenomic peak signals with transcriptomic gene expression, we convert bulk epigenomic data into GAS [13, 14]. For each gene, we aggregate signals from chromatin accessible regions (peaks) within a $\pm 100$ kb window relative to the transcription start site (TSS).

Weights are assigned to peaks based on their genomic distance to the target gene to reflect regulatory potential. Specifically, the weight $w_{p,g}$ for a peak $p$ relative to gene $g$ is defined as: 


(10) \begin{align*}& w_{p,g} = \begin{cases} 1.0 & \mathrm{if}\ d_{p,g} \le 5 \text{ kb (promoter/gene body)} \\ e^{-d_{p,g}/5000} & \mathrm{if}\ 5 \text{ kb} < d_{p,g} \le 100 \text{ kb} \end{cases}\end{align*}

where $d_{p,g}$ is the distance (in bp) from the peak center to the TSS. The decay parameter (5000) scales the distance to kilobases. The final GAS for gene $g$ is calculated as the sum of weighted peak signals, normalized by gene length to mitigate size bias. This transformation effectively maps the sparse epigenomic landscape into a dense pseudo-transcriptomic feature space. Additionally, it does not require the bulk and single-cell data to be generated from paired samples, provided that the reference captures the relevant biological cell states. Specifically, STED requires: (i) an scRNA-seq reference capturing the relevant cell states, (ii) bulk epigenomic profiles in peak or window format, and (iii) a gene annotation for GAS computation. No matched multi-omics from the same biological sample is needed.

#### Inferring cell-type proportions in bulk samples

We estimate cell-type proportions by leveraging the latent topic structure learned by scTopic. First, we establish a probabilistic mapping between topics and cell types using the scRNA-seq reference. The posterior probability of cell type $n$ given topic $k$, denoted $C_{n,k}$, is computed via Bayes’ theorem: 


(11) \begin{align*}& C_{n,k} = P(\text{cell type}_{n} \mid \mathrm{topic}_{k}) = \frac{P(\mathrm{topic}_{k} \mid \text{cell type}_{n}) P(\text{cell type}_{n})}{P(\mathrm{topic}_{k})}\end{align*}

Here, $P(\mathrm{topic}_{k} \mid \text{cell type}_{n})$ represents the average contribution of topic $k$ to cells of type $n$.

Assuming the gene–topic relationship is sufficiently conserved across modalities, we infer the topic composition of the $j$th bulk sample, denoted $\theta ^{(b)}_{\cdot j} = (\theta ^{(b)}_{1j}, \ldots , \theta ^{(b)}_{Kj})$, by applying the pretrained topic model to its GAS profile. The estimated proportion of cell type $n$ in bulk sample $j$ is then 


(12) \begin{align*}& \hat{\pi}_{n,j} = \sum_{k=1}^{K} C_{n,k} \theta^{(b)}_{k,j}\end{align*}

where $\hat{\pi }_{n,j}$ denotes the inferred cell-type proportion. This formulation interprets each bulk epigenomic profile as a mixture of cell types expressed in a shared topic space.

#### Prediction of cell-type-specific epigenetic signals

To infer cell-type-specific regulatory landscapes [e.g. TF binding motifs or super-enhancers (SEs)] from bulk data, we employ a matrix-based reconstruction approach:

1. **Reference expression:** We generate a cell-type $\times $ gene expression matrix $E_{\mathrm{ref}}$ by aggregating raw scRNA-seq counts across cells of the same annotated cell type and applying library-size normalization:
  (13) \begin{align*}& E_{\mathrm{ref}}[c, g] = \frac{\sum_{i \in \mathrm{cells}(c)} X_{i,g}}{s_{c}}, \quad s_{c} = \frac{\sum_{g} \sum_{i \in \mathrm{cells}(c)} X_{i,g}}{10^{4}}\end{align*}
  where $X_{i,g}$ denotes the raw count for gene $g$ in cell $i$, and $s_{c}$ is the library-size scaling factor for cell type $c$ (set to 1 when the total count is zero).
2. **Regulatory map:** A “gene $\times $ peak” weight matrix $W_{\mathrm{gp}}$ is constructed using the distance-decay weights ($w_{p,g}$) defined in the GAS calculation.
3. **Signal inference:** We compute a “cell-type $\times $ peak” potential matrix $S_{\mathrm{cp}}$ via matrix multiplication:
  (14) \begin{align*}& S_{\mathrm{cp}} = E_{\mathrm{ref}} \times W_{\mathrm{gp}}\end{align*}

By combining the inferred cell-type proportions $\hat{\pi }_{n,j}$ with this potential matrix, we decompose the bulk signal into cell-type-specific peak activity profiles, enabling downstream motif enrichment and regulatory element identification. In this framework, SE calling is a downstream analysis performed on reconstructed H3K27ac-like signals rather than an intrinsic component of the topic model itself.

#### Notation summary

For clarity, the main variables used throughout STED are summarized in Table 1.

**Table 1:** Summary of notation used throughout the STED framework

Symbol	Definition	Section
$X_{G}$	Expression variables for a selected gene set $G$	CorEx
$\rho _{g,k}$	CorEx gene–topic soft assignment parameter	CorEx
$\lambda ^{(t)}$	CorEx softmax sharpness parameter	CorEx
$T_{d,i}$	LDA topic assignment for the $i$th gene token in cell $d$	LDA
$\alpha _{k}$	LDA Dirichlet prior on topic proportions per cell	LDA
$\beta _{g}$	LDA Dirichlet prior on gene–topic distributions	LDA
$L_{k,v}$	LDA anchor gene guidance weight	LDA
$W^{(g)}$	Gene–topic matrix ($G \times K$), shared across backends	Unified
$\Theta ^{(sc)}$	Cell–topic matrix ($K \times M$) in the scRNA-seq reference	Unified
$C_{n,k}$	Topic–cell-type association, $P(\mathrm{cell\ type}_{n} \mid \mathrm{topic}_{k})$	Unified
$\theta ^{(b)}_{k,j}$	Inferred topic composition of bulk sample $j$	epiDecon
$\hat{\pi }_{n,j}$	Estimated proportion of cell type $n$ in bulk sample $j$	epiDecon
$E_{\mathrm{ref}}$	Cell-type-aggregated and library-normalized reference expression matrix	epiDecon
$W_{\mathrm{gp}}$	Gene-to-peak weight matrix	epiDecon
$S_{\mathrm{cp}}$	Reconstructed cell-type–peak potential matrix	epiDecon
$w_{p,g}$	Peak–gene distance-decay weight	GAS

### Downstream epigenomic analysis

**Differential peak analysis and annotation:** Differential accessibility was analyzed using *DESeq2* on scaled pseudo-counts. Significant peaks ($|\log _{2}\mathrm{FC}| \ge 1$, adjusted $P <.05$) were annotated to the nearest gene ($\pm 2.5$ kb TSS) using *ChIPseeker*, followed by enrichment analysis via *clusterProfiler* and *msigdbr*.

**Motif enrichment:** TF motif enrichment in differentially accessible peaks was assessed using *HOMER* with standard parameters.

**Super-enhancer identification:** SEs were identified downstream of STED by stitching proximal peaks within 12.5 kb (i.e. min.gapwidth = 12500 in GenomicRanges::reduce), followed by ranking of reconstructed H3K27ac signals. The inflection point is determined by sliding a diagonal line from the maximum signal to the origin along the ranked distribution and identifying the tangent point that minimizes the number of regions below the line, following the ROSE algorithm. All peaks with signals above this inflection point are classified as SEs. For tumor analysis, SEs were lifted to hg38, quantified via *featureCounts*, and analyzed for differential activity using *DESeq2*.

### Benchmarking and evaluation metrics

**Deconvolution accuracy:** Performance was evaluated using PCC and RMSE comparing predicted versus ground-truth values for both cell-type proportions and cell-type-specific signal profiles.

**Comparative analysis:** STED was benchmarked against EPI-ATAC [9] and DeconPeaker [10]. Given that EPI-ATAC relies on a built-in set of cell-type-specific signature peaks optimized for PBMCs, we utilized this dataset to evaluate performance across three distinct feature selection strategies: (i) RNA: Cell-type-specific differential expression genes (DEGs) from scRNA-seq (STED’s standard cross-modal workflow); (ii) ATAC: Differential peaks (DPs) derived directly from the 10x scATAC-seq data; and (iii) ATAC sig: The preselected high-confidence signature peaks from EPI-ATAC.

**Ablation study and robustness analysis:** To evaluate STED’s core components, we compared Full STED against two baselines: (i) No-topic baseline (DirectEref), using non-negative least squares to directly project bulk GAS profiles onto the cell-type reference; and (ii) No-GAS baseline, using raw peak counts instead of cross-modal GAS transformations. For statistical rigor, we performed 1000 bootstrap resamples of the single-cell reference populations. This ensures robust performance estimation (Mean $\pm $ Bootstrap SD) and confirms that observed gains are statistically significant and unbiased.

### Data preprocessing and ground-truth generation

**Single-cell and pseudo-bulk processing:** Ground-truth labels were derived from 10$\times $ Multiome or scCUT&Tag data using standard pipelines (*Scanpy*, *SIMBA*). For scCUT&Tag, we compared two distinct strategies to extract signals: (i) Window-based binning, which quantifies signal intensity across fixed-size genomic windows (50 kb) to mitigate sparsity; and (ii) Peak-centric annotation, which extracts signals exclusively from predefined peak regions.

**Bulk data processing:** Bulk epigenomic data were processed via *bwa-mem2* (alignment), *GATK* (filtering), and *MACS2* (peak calling, --broad), with quantification via *featureCounts*.

**Anchor gene selection:** We employed two sets of anchor genes. Anchor 1 was derived from *CellMarker* (http://117.50.127.228/CellMarker/). Anchor 2 was obtained by identifying statistically significant marker genes for each cell type using the Wilcoxon rank-sum test implemented in the rank_genes_groups function in *Scanpy*.

## Results

### Single-cell topic modeling and epigenetic deconvolution resolves the hierarchical architecture of cellular identity through anchor-guided topic modeling

**Figure 2:**
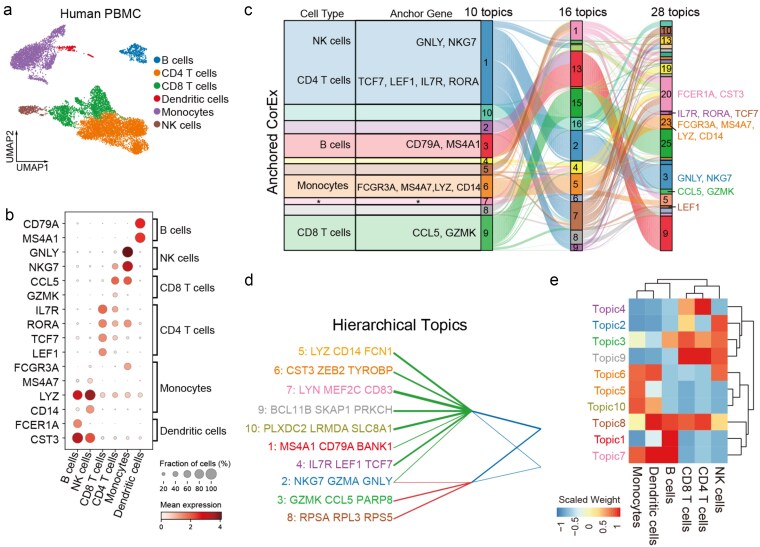
STED extracts biologically interpretable hierarchical topics via anchored genes. Using human PBMC scRNA-seq data visualized in UMAP space (a), STED leverages canonical marker genes as anchors (Anchor 2) (b) to guide biologically meaningful topic discovery. The framework effectively maps these anchor genes to latent topics across varying numbers of topics ($k$), as illustrated by Sankey diagrams for STED-CorEx (c), which link anchor genes to their corresponding cell types. Furthermore, scTopic resolves the hierarchical regulatory structure of cell identities through clustering of the inferred topics, annotated with top representative genes for STED-CorEx (d). This interpretability is further validated by heatmaps quantifying the specific enrichment of inferred topics within annotated cell types for STED-CorEx (e).

We validated scTopic across three datasets (human PBMCs, jejunum, and mouse brain) and utilized comprehensive metrics—including topic coherence, mutual information, and the elbow point of accuracy curves—to guide optimal hyperparameter selection ($k$) (Fig. S1A–E). By incorporating anchor genes (Table S1) to constrain gene–topic associations, scTopic consistently identified biologically coherent topics across diverse resolutions (Fig. 2a and b).

Using PBMCs (Fig. 2a and b) as a primary case study, we visualized the stability and hierarchy of learned topics. Sankey diagrams revealed that genes assigned to specific biological functions remained co-clustered across varying $k$ values (Fig. 2c). scTopic further resolves the hierarchical relationships among these topics through clustering and annotation with top representative genes. Functionally related topics clustered proximally: e.g. CorEx grouped cytotoxic NK/CD8$^{+}$ T cell signatures (*NKG7*, *GZMA*, and *GNLY*) and effector CD8$^{+}$ T cell markers (*GZMK* and *CCL5*) into a unified branch (Fig. 2d).

We established a systematic interpretation framework linking core genes to biological pathways and cell types (Fig. S1E and F). In the PBMC analysis, scTopic distinguished broad myeloid processes (Topic 6: cytokine production in DCs/monocytes) from specialized antibacterial responses (Topic 5: *IL-12* release in monocytes) (Fig. S1G and H). Collectively, these results demonstrate that STED robustly extracts interpretable, hierarchically structured cellular features across diverse biological contexts.

### Single-cell topic modeling and epigenetic deconvolution leverages pretrained models to extract biologically interpretable hierarchical topics

STED pioneers the adaptation of the BERTopic framework—originally designed for natural language processing—to single-cell transcriptomics. By treating cells as “documents” and repurposing semantic embeddings from single-cell foundation models (e.g. scFoundation and scBERT), STED captures high-dimensional biological contexts beyond simple expression counts.

We benchmarked the efficacy of these semantic embeddings against traditional PCA-based clustering. Notably, scFoundation embeddings—without any supervised fine-tuning—achieved cell-type discrimination comparable to PCA across various resolutions, demonstrating remarkable zero-shot generalization. In contrast, scBERT encoder embeddings, even after fine-tuning, showed limited capacity for unsupervised clustering, likely due to model size constraints (Fig. S2). Consequently, scFoundation was prioritized as the semantic backend for STED.

**Figure 3:**
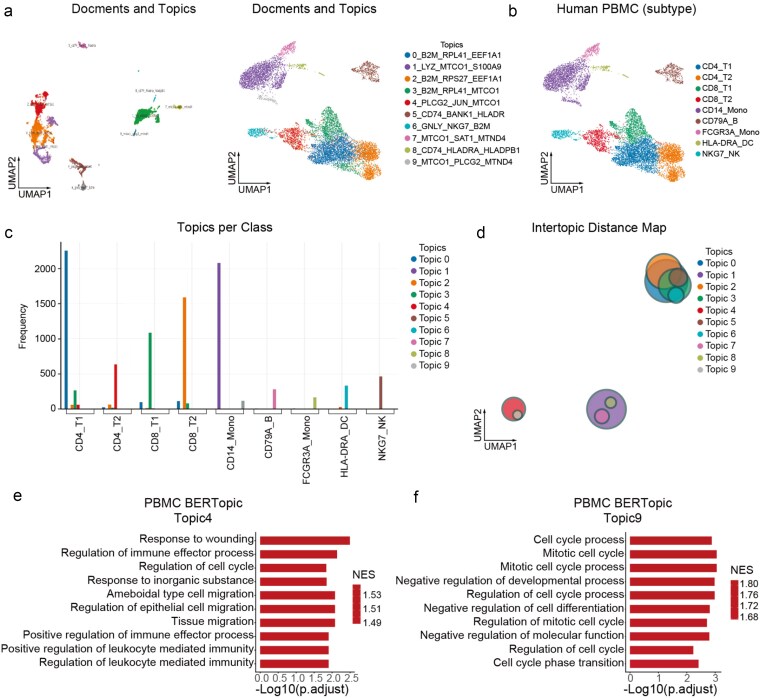
STED leverages the pretrained scFoundation model to extract biologically interpretable hierarchical topics. Utilizing embeddings generated by scFoundation (left) versus PCA (right), STED-BERTopic clusters human PBMC cells into distinct topics visualized in UMAP space (a). These identified topics align closely with biological reality when compared with UMAP visualizations grouped by annotated cell subtypes (b). This alignment is further quantified by bar plots illustrating the correspondence between topics and cell subtypes (c), showing the specific composition of subtypes within each topic. Additionally, the relationship among the topics themselves is resolved through UMAP visualization of their c-TF-IDF representations (d). The biological interpretability of these topics is validated by GSEA, which highlights significant normalized enrichment scores (NES) for genes associated with Topic 4 (e) and Topic 9 (f).

Applying this adapted foundation-model-based framework to human PBMCs revealed a high concordance between latent topics and annotated cell types (Fig. 3a–c). Beyond discrete classification, the model captured biological continua; for instance, CD4$^{+}$ and CD8$^{+}$ T cell subtypes spanned multiple topics, reflecting phenotypic plasticity. UMAP visualization of topic-specific c-TF-IDF representations confirmed that functionally related populations (e.g. monocytes and DCs) clustered together, distinct from the lymphoid lineage (Fig. 3d).

Crucially, STED uncovered transitional and rare states often obscured in standard annotations. For example, Topic 4, associated with an ambiguous CD8$^{+}$ T cell subset (CD8 T2), was enriched for effector differentiation markers, resolving its functional identity (Fig. 3e). Furthermore, Topic 9, which lacked a direct cell-type label, was significantly enriched for cell-cycle regulators (Fig. 3f), successfully identifying a cycling myeloid cell population. These findings demonstrate that translating semantic topic modeling to single-cell data not only validates annotations but also uncovers subtle biological heterogeneity bridging computational semantics and biological discovery.

### Single-cell topic modeling and epigenetic deconvolution achieves robust deconvolution and signal reconstruction across diverse epigenomic modalities

We first evaluated the impact of topic modeling strategies and hyperparameter choices using two sets of anchor genes (Table S1). Minimum topic numbers were initialized based on cell-type diversity for CorEx and LDA, while scFoundation embeddings and parameter combinations were tested for BERTopic.

We benchmarked the deconvolution performance of STED on multi-omics datasets (PBMCs, jejunum, and mouse brain) by aggregating scATAC-seq data into pseudo-bulk profiles. While all modules achieved robust performance (PCC $> 0.8$), CorEx consistently outperformed LDA in both accuracy (Fig. S3) and computational efficiency (Fig. S5A–D). BERTopic exhibited high sensitivity to clustering parameters, achieving optimal performance when topic numbers aligned with cell-type complexity (Fig. S4). For instance, in human jejunum data, a high resolution (partitioning cells into $>100$ topics) yielded excellent results. Since clustering constitutes the primary computational bottleneck for BERTopic, increasing resolution did not significantly incur additional time costs (Fig. S5E and F). Notably, semi-supervised learning with anchor genes enhanced performance in datasets with distinct cell types (e.g. PBMCs) but showed diminishing returns in high-resolution annotations where markers were confounded across subtypes. Furthermore, anchor genes yielded no performance gain for BERTopic (Table 2).

**Table 2:** PCC and RMSE (Mean $\pm $ SE) for topics selected by best PCC and best RMSE

		Selected by PCC			Selected by RMSE		
Model	Anchor set	Topic number/Parameters	PCC	RMSE	Topic number/Parameters	PCC	RMSE
**Dataset 1: Human PBMC (Main)**							
STED-CorEx	Anchors 1	21	**0.988 $\pm $ 0.004**	0.028 $\pm $ 0.005	21	0.988 $\pm $ 0.004	**0.028 $\pm $ 0.005**
STED-LDA	Anchors 1	11	0.972 $\pm $ 0.011	0.072 $\pm $ 0.015	11	0.972 $\pm $ 0.011	0.072 $\pm $ 0.015
STED-CorEx	Anchors 2	25	0.980 $\pm $ 0.005	0.036 $\pm $ 0.003	29	0.979 $\pm $ 0.008	**0.033 $\pm $ 0.006**
STED-LDA	Anchors 2	22	**0.983 $\pm $ 0.005**	0.057 $\pm $ 0.007	22	0.983 $\pm $ 0.005	0.057 $\pm $ 0.007
STED-CorEx	No Anchor	19	**0.984 $\pm $ 0.003**	0.032 $\pm $ 0.004	19	0.984 $\pm $ 0.003	**0.032 $\pm $ 0.004**
STED-LDA	No Anchor	6	0.944 $\pm $ 0.031	0.053 $\pm $ 0.014	6	0.944 $\pm $ 0.031	0.053 $\pm $ 0.014
STED-BERTopic	No Anchor	a5, 0.5	0.928 $\pm $ 0.013	0.069 $\pm $ 0.003	a5, 1	0.927 $\pm $ 0.007	0.065 $\pm $ 0.002
**Dataset 2: Human PBMC (Subtype)**							
STED-CorEx	Anchors 1	28	**0.974 $\pm $ 0.004**	0.023 $\pm $ 0.002	28	0.974 $\pm $ 0.004	**0.023 $\pm $ 0.002**
STED-LDA	Anchors 1	27	0.945 $\pm $ 0.011	0.040 $\pm $ 0.004	27	0.945 $\pm $ 0.011	0.040 $\pm $ 0.004
STED-CorEx	Anchors 2	30	**0.967 $\pm $ 0.011**	0.026 $\pm $ 0.004	30	0.967 $\pm $ 0.011	**0.026 $\pm $ 0.004**
STED-LDA	Anchors 2	19	0.948 $\pm $ 0.006	0.049 $\pm $ 0.004	20	0.917 $\pm $ 0.024	0.043 $\pm $ 0.008
STED-CorEx	No Anchor	23	**0.968 $\pm $ 0.005**	0.023 $\pm $ 0.002	23	0.968 $\pm $ 0.005	**0.023 $\pm $ 0.002**
STED-LDA	No Anchor	25	0.899 $\pm $ 0.016	0.039 $\pm $ 0.002	25	0.899 $\pm $ 0.016	0.039 $\pm $ 0.002
STED-BERTopic	No Anchor	a5, 0.5	0.894 $\pm $ 0.018	0.049 $\pm $ 0.002	a5, 1.2	0.865 $\pm $ 0.014	0.045 $\pm $ 0.002
**Dataset 3: Human jejunum**							
STED-CorEx	Anchors 1	28	**0.923 $\pm $ 0.014**	0.026 $\pm $ 0.002	34	0.920 $\pm $ 0.012	0.025 $\pm $ 0.001
STED-LDA	Anchors 1	28	0.871 $\pm $ 0.048	0.049 $\pm $ 0.005	33	0.851 $\pm $ 0.004	0.047 $\pm $ 0.003
STED-CorEx	Anchors 2	24	0.915 $\pm $ 0.014	0.026 $\pm $ 0.002	32	0.907 $\pm $ 0.007	0.028 $\pm $ 0.002
STED-LDA	Anchors 2	36	0.889 $\pm $ 0.024	0.035 $\pm $ 0.008	35	0.840 $\pm $ 0.006	0.050 $\pm $ 0.003
STED-CorEx	No Anchor	27	**0.928 $\pm $ 0.009**	0.026 $\pm $ 0.002	27	0.928 $\pm $ 0.009	0.026 $\pm $ 0.001
STED-LDA	No Anchor	21	0.781 $\pm $ 0.040	0.052 $\pm $ 0.004	21	0.781 $\pm $ 0.040	0.064 $\pm $ 0.003
STED-BERTopic	No Anchor	f1, 12	0.923 $\pm $ 0.006	0.024 $\pm $ 0.001	f1, 15	0.923 $\pm $ 0.006	**0.024 $\pm $ 0.001**
**Dataset 4: Mouse brain**							
STED-CorEx	Anchors 1	19	**0.931 $\pm $ 0.016**	0.026 $\pm $ 0.003	19	0.931 $\pm $ 0.016	**0.026 $\pm $ 0.004**
STED-LDA	Anchors 1	20	0.839 $\pm $ 0.042	0.042 $\pm $ 0.009	32	0.808 $\pm $ 0.026	0.055 $\pm $ 0.004
STED-CorEx	Anchors 2	19	**0.918 $\pm $ 0.013**	0.027 $\pm $ 0.002	19	0.918 $\pm $ 0.013	**0.027 $\pm $ 0.002**
STED-LDA	Anchors 2	38	0.872 $\pm $ 0.026	0.044 $\pm $ 0.007	42	0.855 $\pm $ 0.015	0.045 $\pm $ 0.003
STED-CorEx	No Anchor	16	**0.913 $\pm $ 0.011**	0.031 $\pm $ 0.002	16	0.913 $\pm $ 0.011	**0.031 $\pm $ 0.003**
STED-LDA	No Anchor	35	0.900 $\pm $ 0.044	0.024 $\pm $ 0.006	35	0.900 $\pm $ 0.044	0.054 $\pm $ 0.003

Note: Topics were selected by maximizing PCC (Selected by PCC) or minimizing RMSE (Selected by RMSE). Values represent Mean $\pm $ SE. Topic ranges: 6–30 (PBMC), 12–36 (Jejunum), 16–48 (Mouse Brain). For STED-BERTopic, parameters are resolution, and target high resolution. Bold values indicate the best performance for each metric.

**Figure 4:**
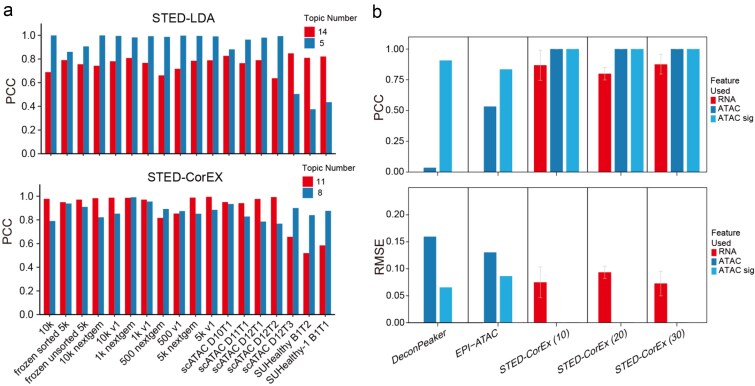
Performance comparison against existing methods. STED is evaluated against ground truth using independent scRNA-seq reference datasets across varying numbers of topics (a), with results displayed for LDA (upper panels) and CorEx (lower panels). Building on this, STED (implemented with CorEx) is benchmarked against DeconPeaker and EPI-ATAC to assess performance stability (b). This comparison spans three distinct feature selection strategies: cell-type-specific DEGs identified from scRNA-seq via Wilcoxon rank-sum test (RNA), differential peaks derived from scATAC-seq (ATAC), and high-confidence internal signature peaks from the EPI-ATAC framework (ATAC sig).

To evaluate robustness against reference heterogeneity, we applied STED to pseudo-bulk ATAC-seq profiles derived from a 10$\times $ Multi-omics PBMC dataset (10K cells) using diverse human PBMC references. When deconvolution was performed using mismatched references (e.g. different sequencing platforms), CorEx exhibited superior stability compared with LDA (Fig. 4a), indicating that its information-theoretic objective is more resilient to batch effects.

To evaluate the synergistic contributions of scTopic and epiDecon, systematic ablation studies were performed on PBMC, mouse brain, and human jejunum datasets (Table 3). Full STED consistently outperformed all baselines, demonstrating robust accuracy (PCC $> 0.9$) and low error rates. Conversely, removing either topic modeling (No-topic) or cross-modal transformation (No-GAS) significantly degraded performance across all tissues. The limited efficacy of the No-topic baseline indicates that direct projection inadequately addresses the sparsity and noise inherent to scRNA-seq references. Furthermore, the underperformance of the No-GAS baseline highlights the necessity of a biophysical bridge for aligning chromatin accessibility with cellular identity. These results confirm that integrating latent regulatory modeling with physics-based signal translation is essential for effective cross-modal deconvolution.

**Table 3:** Ablation experiment results across multiple datasets (Mean $\pm $ Bootstrap Std)

Dataset	Condition	PCC	RMSE
PBMC	**Full STED**	**0.993** $\pm $ **0.019**	**0.026** $\pm $ **0.009**
	No-Topic	$-0.719$ $\pm $ 0.214	0.272 $\pm $ 0.053
	No-GAS	$0.132$ $\pm $ 0.471	0.219 $\pm $ 0.061
Brain	**Full STED**	**0.895** $\pm $ **0.070**	**0.031** $\pm $ **0.006**
	No-Topic	$-0.375$ $\pm $ 0.141	0.141 $\pm $ 0.027
	No-GAS	$-0.264$ $\pm $ 0.201	0.142 $\pm $ 0.026
Jejunum	**Full STED**	**0.911** $\pm $ **0.069**	**0.028** $\pm $ **0.007**
	No-Topic	$-0.086$ $\pm $ 0.379	0.105 $\pm $ 0.018
	No-GAS	$0.274$ $\pm $ 0.349	0.117 $\pm $ 0.021

Note: Results represent point estimates $\pm $ bootstrap standard deviation (1000 resamples of cell types). Full STED means STED-CorEx. Topic dimensions ($K$) are 15 for PBMC and 20 for Brain/Jejunum. Bold values denote the highest performance for each evaluation metric.

STED was also benchmarked against two bulk ATAC-seq deconvolution tools, EPI-ATAC [9] and DeconPeaker [10]. STED demonstrated remarkable versatility, achieving high accuracy (PCC $\approx $ 1) across diverse feature sets (Fig. 4b). In both mouse brain and human jejunum datasets, STED achieved consistent and comparable results using either DPs or DEGs (Fig. S6). In contrast, competing methods relied heavily on predefined signature peaks and failed in non-standard tissues like the mouse brain.

**Figure 5:**
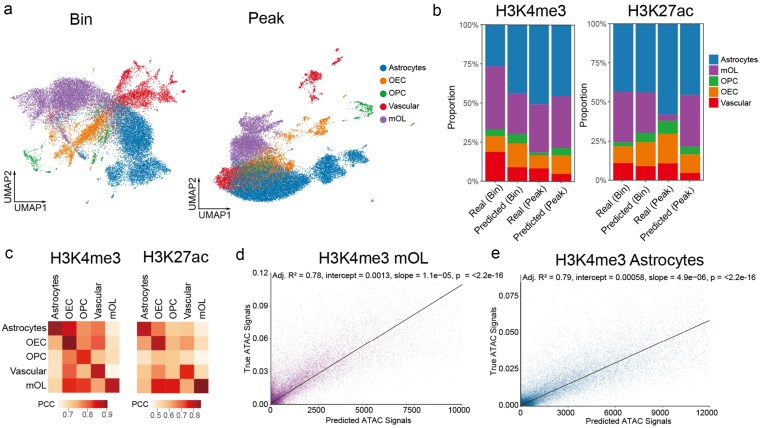
STED’s application to scCUT&TAG data. To evaluate performance on scCUT&TAG profiles, integrated mouse brain H3K27ac, H3K4me3, and RNA data are visualized in UMAP space (a), comparing window-based (50 kb) versus peak-centric integration strategies across cell types (mOL, mature oligodendrocytes; OEC, olfactory ensheathing cells; OPC, oligodendrocyte progenitor cells). The accuracy of STED is demonstrated by the close agreement between predicted and ground-truth cell-type proportions for both H3K4me3 and H3K27ac data (b). This concordance is further validated globally by heatmaps displaying the Pearson correlation coefficient (PCC) between simulated and ground-truth peak signals across all cell types (c), and specifically highlighted through scatter plots showing high correlation for mOL (d) and astrocytes (e) in H3K4me3 data. Model fit is quantified by the adjusted R-squared (adj. $R^{2}$).

Expanding to histone modifications, we applied STED to paired scCUT&Tag data (H3K4me3 and H3K27ac) from the mouse brain [7], comparing window-based binning (50 kb) versus peak-centric integration. Window-based strategies yielded superior consistency, particularly for sparse H3K27ac data where peak-centric approaches failed to annotate rare populations (e.g. mature oligodendrocytes) due to incomplete peak calling (Fig. 5a and b). Reconstructed cell-type-specific signals demonstrated high concordance with ground-truth single-cell profiles (Fig. 5c–e), validating STED’s utility for interpreting bulk histone modifications.

### Single-cell topic modeling and epigenetic deconvolution captures epigenetic heterogeneity across cell types in real-world bulk data

To evaluate the performance of STED in real-world biological contexts, we applied it to a hematopoietic stem cell (HSC) differentiation system [15] and zebrafish inner ear tissues [16].

**Figure 6:**
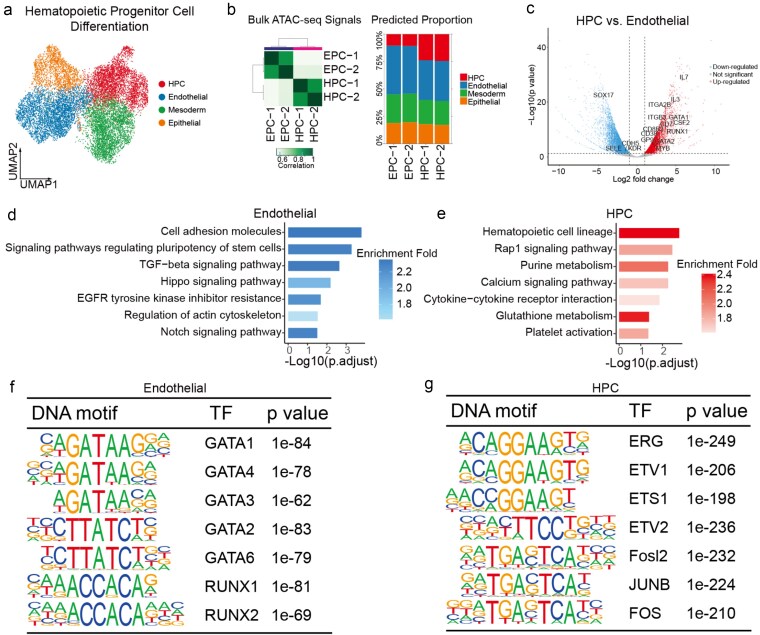
STED reveals epigenetic regulatory mechanisms driving the EHT. Distinct cell populations, including HPCs, are visualized in UMAP space using the EHT dataset (a). The study leverages a differentiation model where human pluripotent stem cells (hPSCs) generate vascular endothelial progenitors (EPCs) and subsequently HPCs, with bulk ATAC-seq data generated at EPC and HPC stages alongside single-cell profiles during the EHT. The reliability of this approach is validated by the high Pearson correlation coefficients between bulk ATAC-seq replicates (b, left) and the consistent cell-type proportions predicted by STED across different developmental stages (b, right). At the molecular level, differential epigenetic landscapes between reconstructed endothelial cells and HPCs are highlighted by a volcano plot of peak signals (c). These distinct peaks map to biologically relevant pathways, as shown by Gene Ontology (GO) enrichment analysis for endothelial-specific (d) and HPC-specific (e) genes. Furthermore, the regulatory mechanisms driving these identities are corroborated by Homer motif analysis, which identifies significantly enriched transcription factor motifs specific to endothelial (f) and HPC (g) predicted peaks.

We first analyzed the endothelial-hematopoietic transition (EHT) to validate STED in a dynamic developmental system (Figs 6a and S7A). STED accurately inferred stage-specific shifts in cellular composition, capturing the marked expansion of hematopoietic progenitor cells (HPCs) during differentiation (Fig. 6b). Beyond proportions, STED reconstructed cell-type-specific chromatin accessibility profiles that recovered $>90\%$ of validated bulk peaks (Fig. S7B and c).

Differential peak analysis successfully identified lineage-specific regulatory landscapes. Endothelial-specific peaks were enriched for cell adhesion and TGF-$\beta $ signaling, while HPC-specific peaks harbored key hematopoietic drivers (e.g. *RUNX1*, GATA family [17]) and platelet activation pathways (Fig. 6c–e). Crucially, STED demonstrated superior sensitivity over raw scATAC-seq data. Key regulators like *CD38* and *RUNX1*, which exhibited weak differential signals in sparse single-cell data (Fig. S7D and E), were robustly recovered by STED through reference-based deconvolution of deep-sequenced bulk data. Motif enrichment analysis further confirmed high concordance with single-cell regulatory topics inferred by SCENIC+, identifying distinct lineage-defining factors (ETS/FOS in endothelial cells versus GATA/RUNX1 in HPCs) (Figs 6f–g and S7F–G).

To further assess the framework’s robustness in other organisms, we applied STED to zebrafish inner ear data [16]. STED accurately recapitulated cell-type proportions in bulk ATAC-seq profiles, confirming that utricle and saccule tissues exhibit compositions consistent with ground-truth single-cell annotations from the original study (Fig. S8). These results, combined with the HSC analysis, confirm that STED effectively resolves biologically meaningful regulatory dynamics across diverse species and developmental contexts.

### Single-cell topic modeling and epigenetic deconvolution enables the identification of cell-type-specific super-enhancers from bulk data

Leveraging STED’s capacity to reconstruct cell-type-specific H3K27ac signals (Fig. S9), we investigated SEs in colorectal cancer (CRC) using paired single-cell [18] and bulk ChIP-seq data [19]. We reconstructed SE landscapes for tumor epithelial cells and normal enterocytes, identifying 578 and 386 SEs, respectively (Fig. S10A and B).

Analysis of tumor-specific SEs revealed a dual signature of lineage retention and pathogenic deregulation. While tumor cells maintained an epithelial core regulatory circuitry (e.g. *CDX1* and *KLF5*), they exhibited aberrant hyperactivation of the Wnt/$\beta $-catenin pathway, evidenced by tumor-exclusive SEs near *AXIN2*, *RNF43*, and *ASCL2* (Fig. S10c). Functional enrichment further indicated metabolic reprogramming: tumor SEs prioritized protein biosynthesis (e.g. ER targeting) and glycolysis (Warburg effect) to support rapid proliferation (Fig. S10D).

In stark contrast, normal enterocyte SEs primarily drove physiological homeostasis and lipid metabolism (e.g. fatty acid oxidation). Key targets included structural regulators (*EPHB3* and *CDHR2*) and transporters (*SLC7A7*), reflecting the functional role of colonocytes in absorption and barrier defense (Fig. S10B and E).

Finally, we compared STED against standard bulk analysis [19]. While bulk methods recovered some drivers, they were heavily confounded by the tumor microenvironment, enriching heterogeneous stromal and immune signatures (see Supplementary Information). STED, by resolving tumor-intrinsic signals, effectively filtered out these artifacts to isolate true epithelial drivers.

## Discussion

The integration of single-cell resolution with the sequencing depth of bulk genomics represents a frontier in systems biology. In this study, we present STED, a unified framework that bridges these modalities not merely through correlation but through shared latent regulatory topics.

The central contribution of STED is that CorEx, LDA, or BERTopic can be embedded into a common cross-modal transfer framework. In STED, these alternative topic-learning strategies are used as interchangeable backends for estimating a shared set of intermediate quantities: gene–topic structure, cell–topic structure, and topic–cell-type associations. This design makes it possible to compare backend behavior within one workflow and, more importantly, to couple transcriptome-derived latent programs with bulk epigenomic deconvolution. We therefore view the methodological novelty of STED as the unification of topic-based latent transfer, GAS-based cross-modal projection, and downstream signal reconstruction within a single modular framework.

This distinction is important when considering why STED is needed beyond a loose combination of existing pipelines. A conventional workflow can separately perform scRNA-seq annotation, bulk deconvolution, and motif analysis, but such pipelines typically stop at abundance estimation or require rigid tissue-specific signature peaks. STED instead uses topic structure as an intermediate representation that is learned from scRNA-seq, projected to bulk epigenomic data, and then reused for reconstruction of cell-type-specific regulatory signals. In practice, this design improves portability across tissues and modalities, and it is especially useful when the biological signal of interest is diluted in bulk profiles by cellular mixing.

A critical methodological advance in STED is the adaptation of the CorEx framework to single-cell omics. Our results indicate that CorEx is often advantageous in sparse transcriptomic settings because its information-theoretic objective captures higher-order gene dependencies without relying on a multinomial count model. LDA remains useful as a classical probabilistic baseline, while BERTopic provides an embedding-based route to discover biologically meaningful programs when semantic cell-state structure is well captured by foundation-model embeddings. By casting these approaches into the same transferable interface, STED separates backend-specific topic discovery from the downstream deconvolution problem.

The ability to move from cell-type proportion estimation to signal reconstruction is another key advantage of STED. Standard deconvolution tools usually end with estimated abundances, whereas STED extends the workflow to reconstruct cell-type-specific peak activity profiles that can support differential peak analysis, motif enrichment, and SE discovery. The GAS step is central to this transition because it provides a modality bridge from peak space to gene-linked regulatory space. Traditional “peak-centric” approaches often fail for broad histone marks (e.g. $H3K27ac$) in single-cell data because sparse reads fail to pass peak-calling thresholds in rare populations. Our window-based GAS is particularly valuable for broad or weak chromatin signals, for rare populations, and for disease samples in which tumor-intrinsic or lineage-restricted programs are masked in the bulk average.

These properties make STED particularly relevant for resolving complex disease-associated epigenetic heterogeneity. In disease contexts where bulk profiles conflate biologically distinct cell populations, topic models address this challenge by identifying co-varying gene modules that are statistically robust to dropout noise and sample-composition shifts; these modules act as de-noised priors for deconvolution. In CRC, e.g. the tumor-intrinsic Wnt/$\beta $-catenin SE program was recovered only after STED’s topic-based decomposition filtered out stromal and immune admixture from the bulk H3K27ac signal. We expect the same principle to apply to other oncogenic, inflammatory, and developmental systems where rare pathogenic cell states or weak regulatory shifts are masked in the bulk average.

Despite these advances, STED faces specific technical constraints. First, performance is sensitive to the compatibility of reference datasets. Our results indicate that mismatched references (e.g. cross-platform variations) can lead to fluctuations in deconvolution accuracy, highlighting the need for context-matched references. Second, the current GAS calculation assumes a positive linearity between epigenetic signals and gene expression. While valid for activating marks (e.g. ATAC-seq, $H3K4me3$,and $H3K27ac$), this assumption may distort repressive landscapes (e.g. $H3K27me3$).

Future work will therefore focus on three directions: improving robustness to reference mismatch, extending the cross-modal transfer function beyond linear activating relationships, and formalizing additional downstream modules for disease-oriented applications. With these extensions, STED could become an even more general framework for extracting cell-type-resolved regulatory information from the extensive repositories of legacy bulk epigenomic data.

**Key Points**

- **Cross-modal innovation**: single-cell topic modeling and epigenetic deconvolution (STED) bridges the gap between transcriptomics and epigenomics, leveraging single-cell RNA references to reconstruct cell-type-specific signals from diverse bulk chromatin profiles (ATAC/ChIP/CUT&Tag).
- **Versatile modeling strategies**: The framework offers flexible options—including information-theoretic CorEx for sparsity handling and foundation-model-based BERTopic for semantic context—to extract interpretable hierarchical topics.
- **Discovery of pathogenic mechanisms**: STED computationally purifies tumor epigenetic landscapes, uncovering tumor-intrinsic super-enhancers in colorectal cancer that were obscured in bulk assays.
- **Democratizing epigenomic analysis**: Unlike tools dependent on rigid atlases, STED utilizes a physics-based gene activity score transfer mechanism, enabling high-resolution re-mining of massive legacy bulk repositories across species.

## Supplementary Material

Supplementary_Files_bbag347

## Data Availability

The source code for the STED Python package is available on GitHub (https://github.com/LiaoYunxi/STED) and archived on Zenodo with the identifier http://doi.org/10.5281/zenodo.17931340 [20]. The demonstration data and supplementary materials supporting the findings of this study are available on Zenodo with the identifier http://doi.org/10.5281/zenodo.17931080 [21]. The publicly available data used for analysis are accessible in the following repositories: 10$\times $ Multiome datasets: Human PBMC, human jejunum, and mouse brain data were obtained from https://www.10xgenomics.com/datasets. Additional 10$\times $ scRNA-seq human PBMC data were sourced from https://github.com/GreenleafLab/ArchR_2020. Mouse brain scCUT&Tag data (GSE163532) [7], zebrafish inner ear data (GSE192947) [16], human HSC differentiation data (GSE168372) [15], single-cell transcriptomic data (GSE132465 and GSE144735) [18], and H3K27ac ChIP-seq data (GSE166254) [19] from primary colorectal tissue and normal mucosa were retrieved from the Gene Expression Omnibus database.
