# Supplementary material for: STED: flexible cross-modal topic modeling infers cell-type-specific regulatory landscapes from bulk epigenomics
Source: Brief Bioinform. 2026 Jun 30;27(3):bbag347. doi: 10.1093/bib/bbag347 (PMC13317755; doi:10.1093/bib/bbag347)
Supplement: Supplementary_Files_bbag347 [file supplementary_files_bbag347.pdf]

Table S1 Consolidated anchor genes for Human PBMC, Human Jejunum, and Mouse Brain cell types.

Cell Type	Anchor 1 (Literature)	Anchor 2 (Data)
**Dataset 1: Human PBMC**		
B cells	BCL7A, FCER2, IGHD, IGHM, PAX5, TCL1A, AIM2, CR2, JCHAIN	CD79A, MS4A1
NK cells	CX3CR1, IL2RB, PTPRC, SELL, FCGR3A, FCGR3B	GNLY, NKG7
CD8 T cells	CD8A, CD8B	CCL5, GZMK
CD4 T cells	CCR4, CD27, CD4, CTLA4, FOXP3, ICOS, IKZF2, IL2RA, CD28, EEF1B2, FHIT, GIMAP5, GIMAP8, PRKCA, RPS5, RSL1D1, SATB1, SLC40A1, SVIL, TESPA1, TSHZ2, CCR6, DPP4	IL7R, RORA, TCF7, LEF1
Monocytes (CD14)	CCR2, CD14, CD68, CD86, FCGR1A, ITGAM, KIT	CD14, LYZ
Monocytes (FCGR3A)	CCR2, FCGR3A, CD68, CD86, FCGR1A, ITGAM, KIT	FCGR3A, MS4A7
Dendritic cells	CD86, CD80, CD83, IL3RA, NRP1, CLEC4C, JCHAIN, LILRA4, MZB1, IL5RA, MS4A3, FCER1A, HPGD, CST3, PLD4, ISG15, UBC, CLEC10A, IRF8, IFITM3, IFI6, CYBA, CD1C, HSPA5, IRF7, CLEC12A, LILRB4, CD1E, THBD	FCER1A, CST3
**Dataset 2: Human Jejunum**		
Myeloid	CD4, S100A9, C1QC, CCDC88B, CCL4, CD14, CD1C, CD63, CLEC10A, CST7, CTSS, HLA-DOB, HLA-DR, IL8, KLRC1, LRRC2, MS4A7, NFKBIA, NKG7, NLRP12, OSM, S100A8, TPSAB1	CD4, DOCK2
Lymphocyte	CXCL13, LAG3, TOX	FYN, IL7R
Fibroblast	COL1A1, COL1A2, DCN, POSTN, WNT4	COL4A2, COL4A1
Stromal	ACTA2, BMP4, CCL11, CD36, CD68, CD90, CDH5, CHI3L1, CLEC9A, DARC, DCN, FAP, MADCAM1, PLVAP, RBP7, RGS5, RSPO3, S100B, SMA, SOX10, SOX17, TAGLN, Vimentin, VWA1, WNT2B, WNT5B, XCR1	APOC3, MYH11
Vascellum	CD36, CDH5, PLVAP	CD36, CDH5
IEC	EPCAM, CDH1, CHGA, CLDN4	CACNA1D, LGR5
Microfold	GP2, TNFAIP2	GP2
Enterocyte	FABP1, ALPI, ANPEP, FABP2, GUCA2A, KRT20	SLC13A2, SLC26A3
Tuft	DCLK1	POU2F3, TRPM5
Goblet	MUC2, SPINK4, FCGBP, SPDEF, BCAS1, CLCA1, ERN2, MUC4	MUC2, FCGBP
Mesothelium	ACTA2, ADAM28, ADAMDEC1, CENPF, GATA6, GINS2, GREM2, HPGD, IL18, MFAP4, MKI67, MYH11, PCLAF, SOX6, STX2, TOP2A, TYMS, WNT10A	CLDN4, PTH2R
Neuroendocrine	CHGA	CHGA, NEUROD1
**Dataset 3: Mouse Brain**		
Astrocyte	Gfap, Aqp4, S100b, Aldh1l1, Slc1a3, Sox9, Slc1a2, Clu, Aldoc, Glul, Mlc1, Atp1b2, Cd44, Gja1, Slc25a19, Slc14a1, Vim	Fgf3, Aldoc
Mature_Oligo	Mbp, Plp1, Mog, Olig2, Mag, Olig1, Cnp, Mobp, Cldn11, Sox10, Pdgfra	Mog, Mag
Early_Oligo	Mbp, Plp1, Mog, Olig2, Mag, Olig1, Cnp, Mobp, Cldn11, Sox10, Pdgfra	Pdgfra, Olig1
Endo-SMC	Cldn5, Cd31, Cd34, Flt1, Vwf	Rgs5, Pecam1
VLMC	Col1a1, Lum	Dcn, Slc47a1
Micro-PVM	Cd206, Cd163, Cd68	Cx3cr1, C1qa
Sst_GABAergic	Gad1, Slc32a1	Sst
Gm10754_GABAergic	Gad1, Slc32a1	Gm10754, Meis2
Lypd1_GABAergic	Gad1, Slc32a1	Lypd1, Meis2
Htr4_GABAergic	Gad1, Slc32a1	Htr4, Gad2
BBB_cells	-	Col8a1, Ocln
Ependymal	-	Tmem212, Dnah11
L6b	-	Nr4a2
L6_Car3	-	Car3, Grik3, Hs3st4
L4/5_Lamp5	-	Lamp5
L2_ENTI	-	Tafa1, Slc17a7

Note: Anchor 1 refers to marker genes derived from literature or standard databases. Anchor 2 refers to marker genes identified in the current dataset.

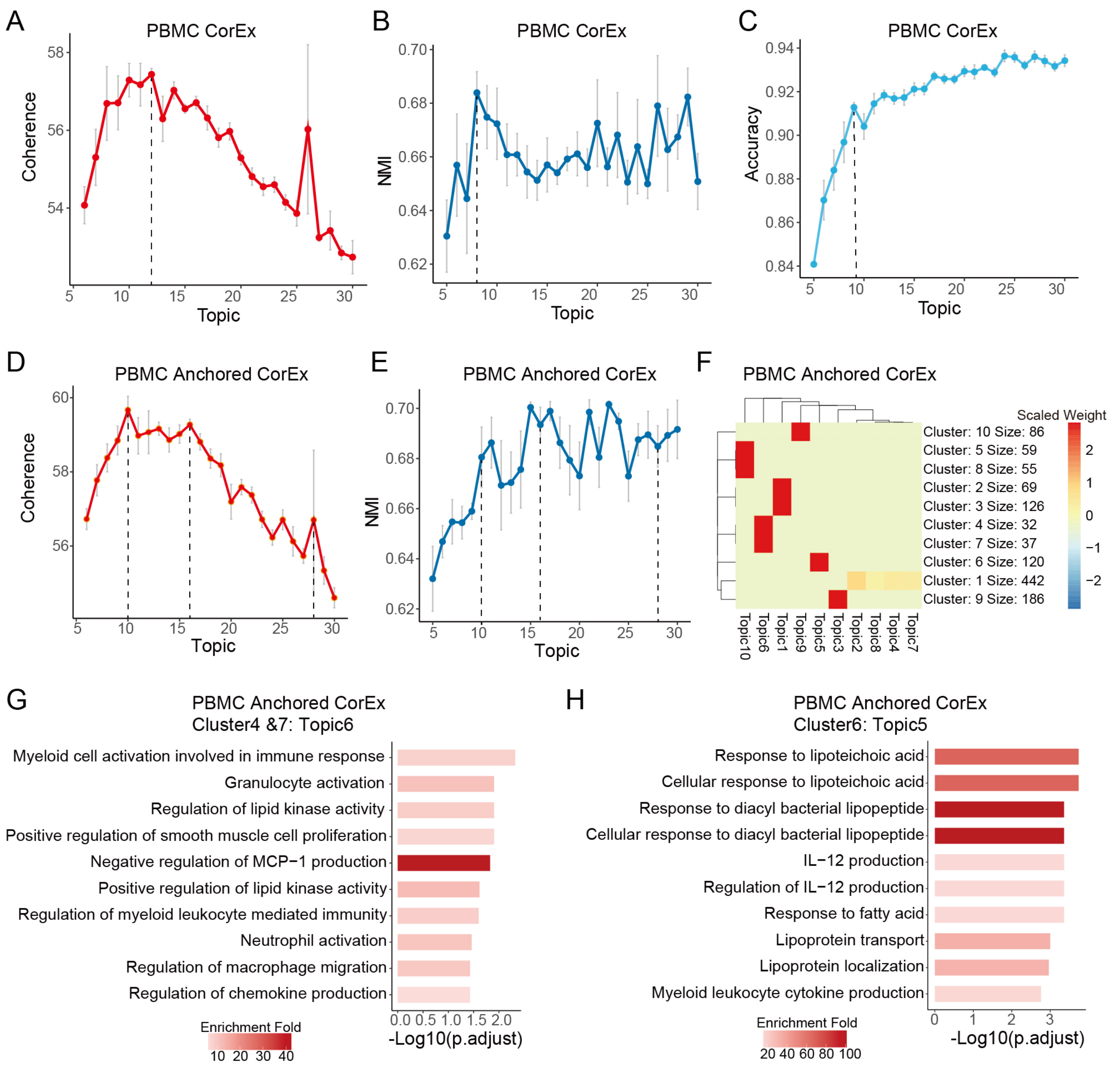

Figure S1 Hyperparameter selection and biological validation of STED. Performance of the STED-CorEx model was evaluated on PBMC data across different topic numbers (k) using topic coherence (**A**), NMI (**B**), and cell type prediction accuracy via linear regression (**C**). Extending this to STED-anchored CorEx, model stability was further assessed via coherence (**D**) and NMI (**E**). The resulting model's biological relevance was demonstrated by clustering the gene-topic distribution (**F**) and identifying enriched GOBP pathways within these topic-associated gene clusters (**G–H**).

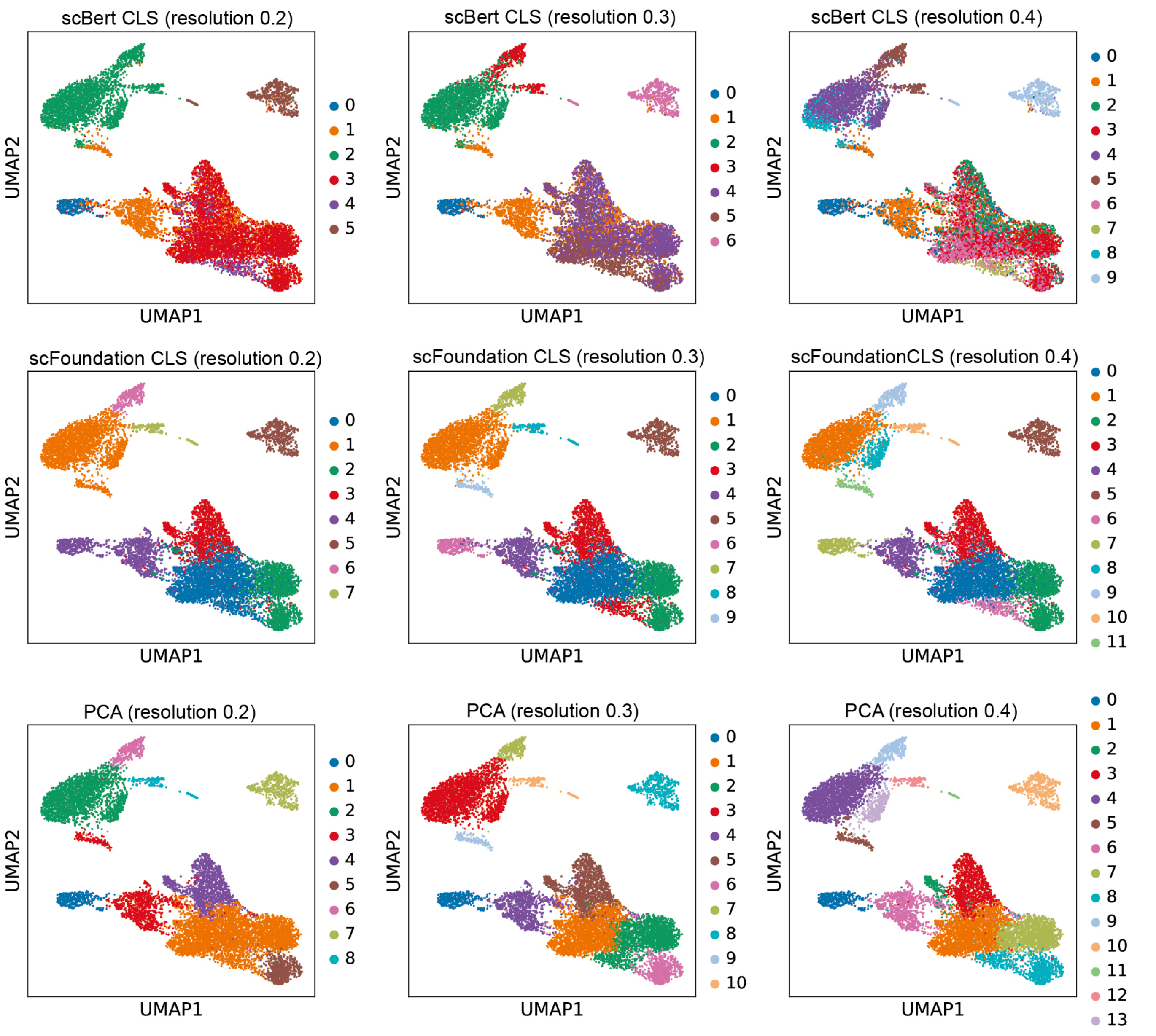

Figure S2 Comparison of clustering performance between PCA-based dimensionality reduction and STED's BERTopic module. Human PBMC scRNA-seq data is visualized in UMAP space across varying cluster resolutions. The analysis contrasts the performance of STED's BERTopic module—utilizing CLS embeddings from scBERT (top) and scFoundation (middle)—against standard PCA-based dimensionality reduction (bottom). This comparison highlights the impact of the underlying embedding model on cluster definition and structural preservation at different granularities.

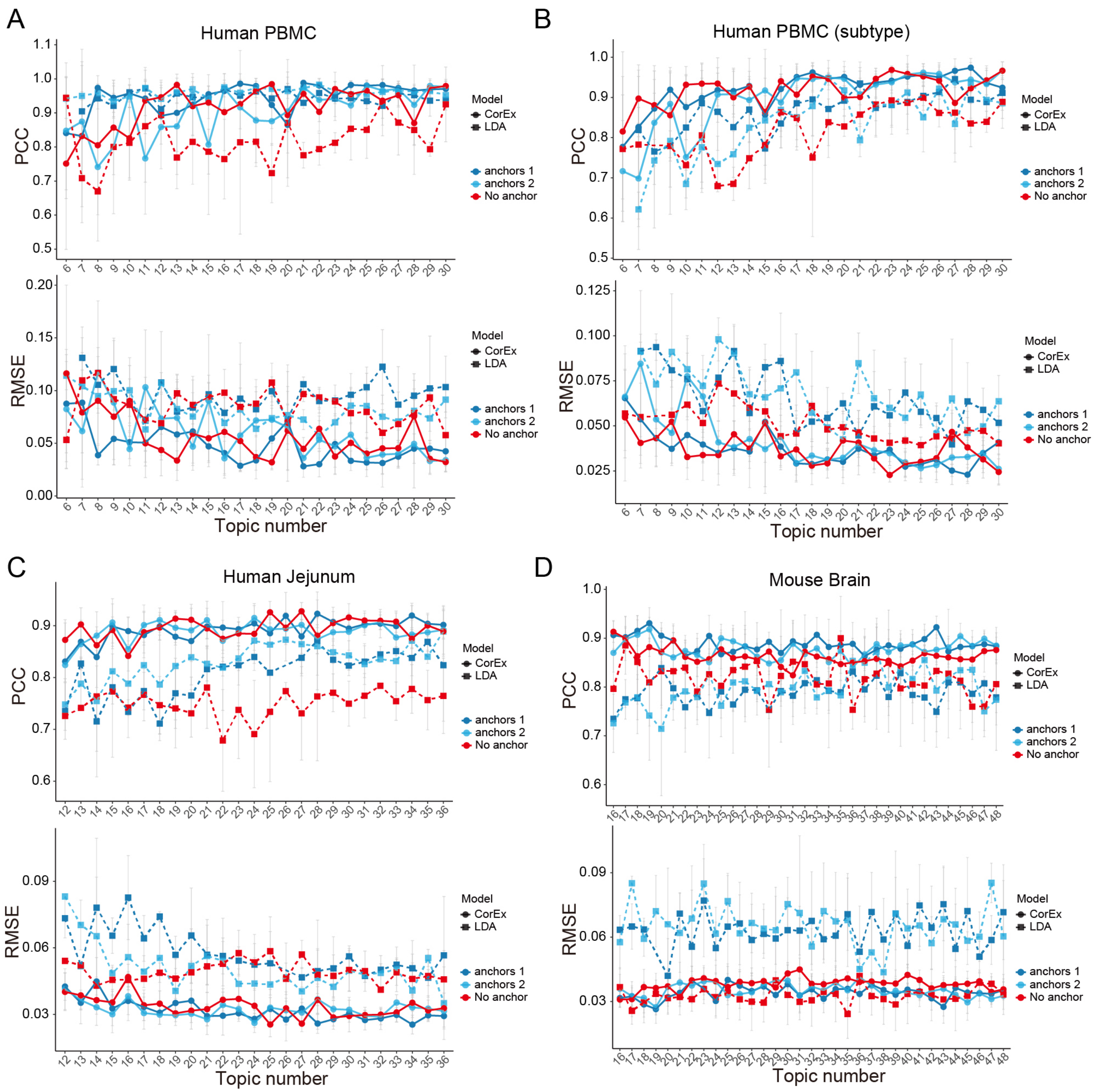

Figure S3 Benchmarking of STED components. To identify the optimal modeling strategy, the performance of Latent Dirichlet Allocation (LDA) and CorEx models is compared using two distinct anchor gene approaches: literature-curated markers (Anchor 1) and differentially expressed genes (Anchor 2). This benchmarking evaluation spans diverse biological contexts, reporting Pearson correlation coefficients (PCC) and Root Mean Square Error (RMSE) for human PBMC (**A**–**B**), human jejunum (**C**), and mouse brain (**D**). These comparisons assess the robustness of different topic modeling and feature selection combinations across varying tissue types.

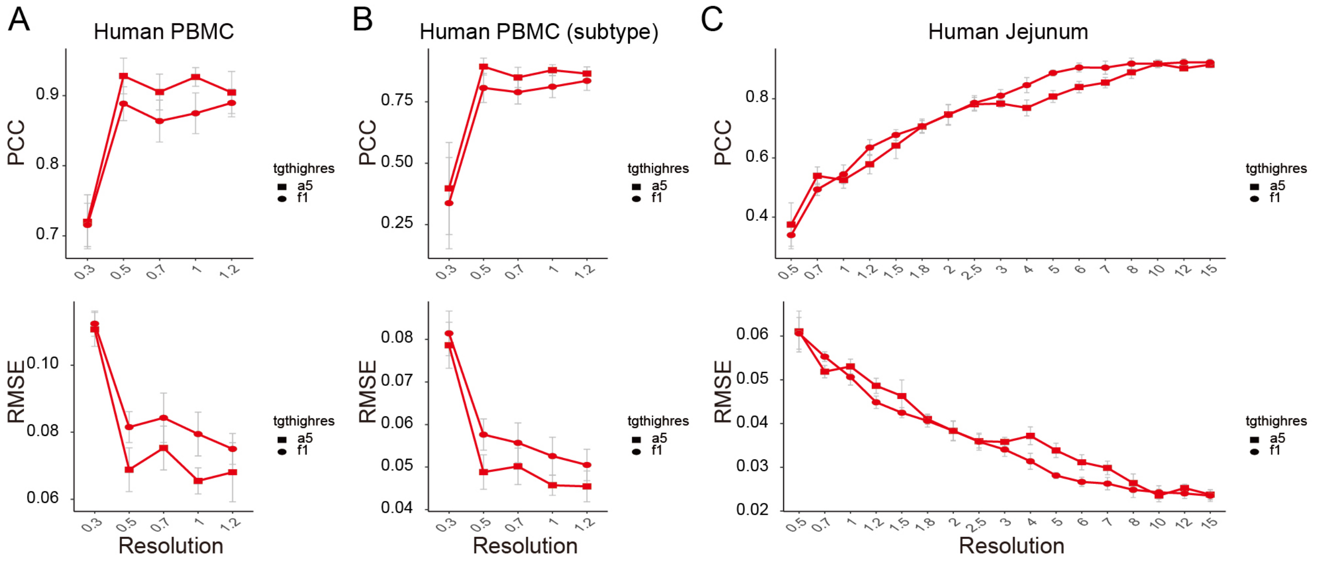

Figure S4 Performance optimization of STED-BERTopic. To identify the optimal configuration for STED-BERTopic, a comprehensive parameter sweep was performed on human PBMC (**A**–**B**) and human jejunum (**C**) datasets. The analysis evaluates the impact of varying combinations of two key hyperparameters: 'resolution', which modulates the granularity of clustering, and 'tgthighres', a crucial scFoundation hyperparameter that controls the target sequencing depth (Total Counts) of the model's output.

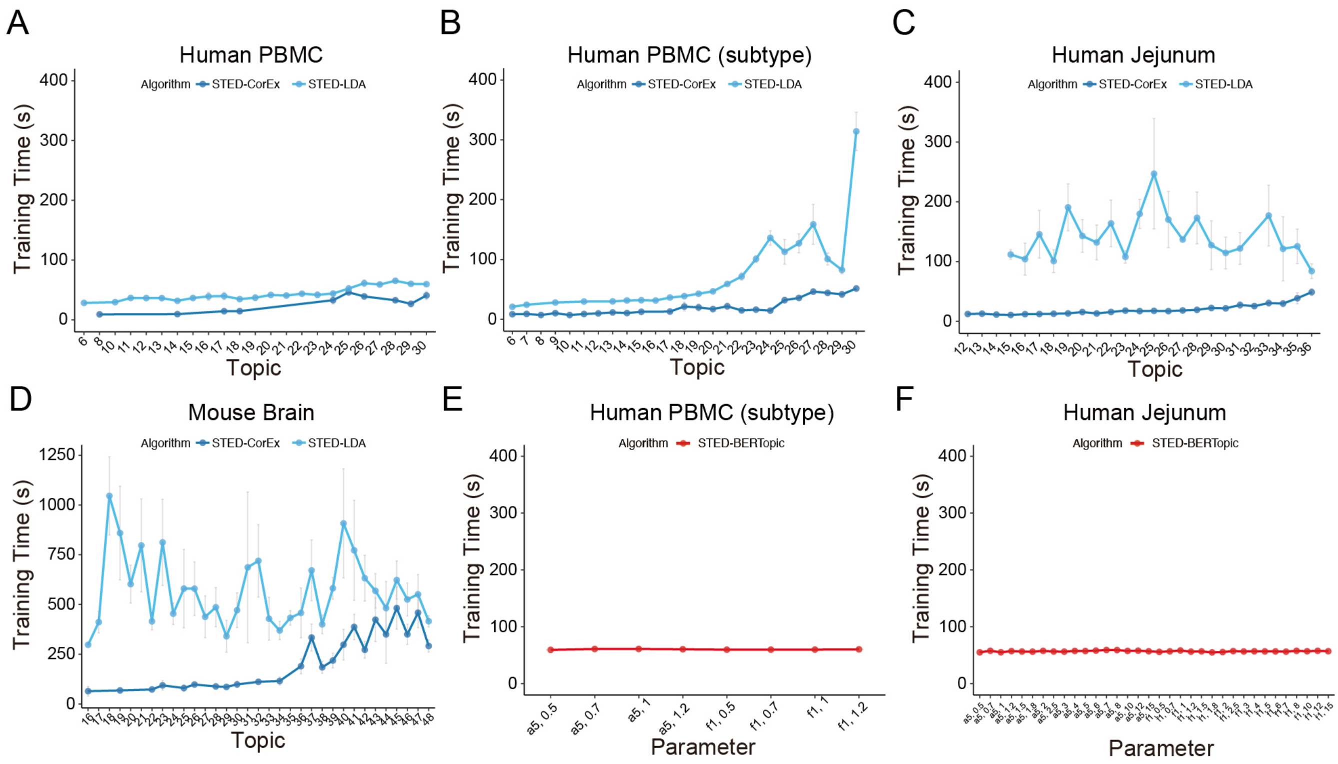

Figure S5 Computational efficiency analysis of STED components. To evaluate the scalability of the framework, the computational runtime of STED-LDA and STED-CorEx models is benchmarked across human PBMC (**A**–**B**), human jejunum (**C**), and mouse brain (**D**) datasets. Additionally, the efficiency of the STED-BERTopic module is assessed on human PBMC (**E**) and human jejunum (**F**). Notably, the reported time for STED-BERTopic excludes the duration required for embedding generation via scFoundation, as this step is contingent upon total cell numbers and available GPU computational resources.

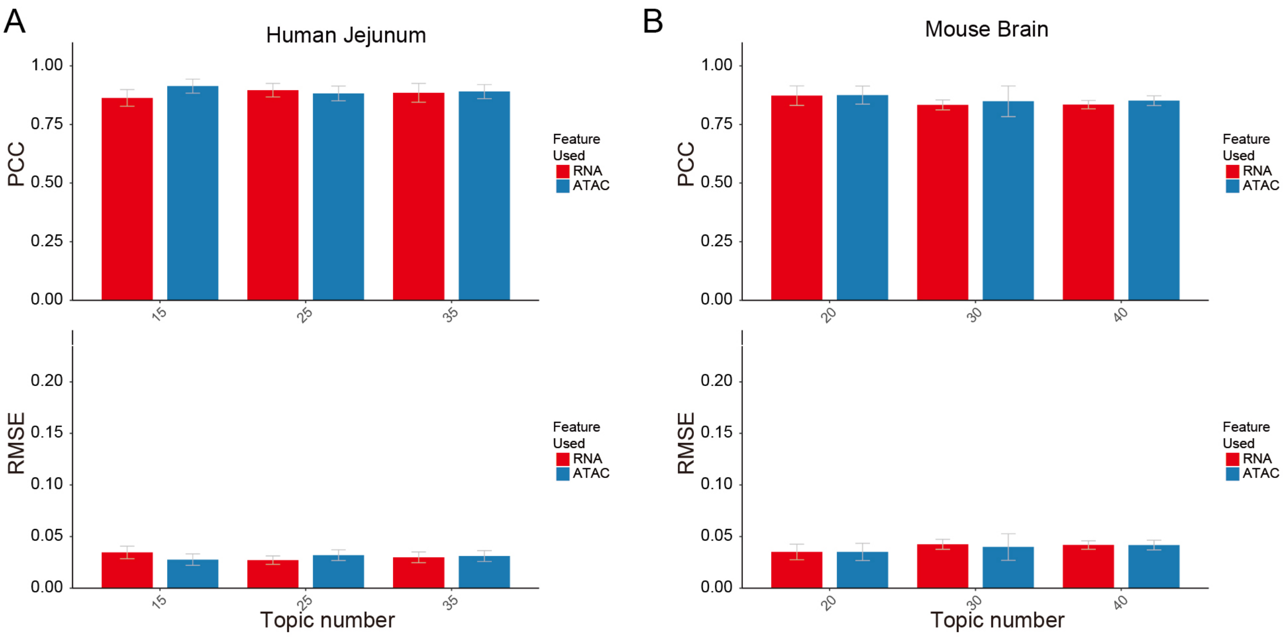

Figure S6 Performance benchmarking of STED across diverse feature selection strategies for topic modeling. Bar plots illustrate the comparative performance of STED (implemented with CorEx) under different feature selection strategies on human Jejunum (**A**) and mouse brain (**B**) datasets. The evaluation contrasts two primary approaches: using cell-type-specific differentially expressed genes identified from scRNA-seq data via Wilcoxon rank-sum test (**RNA**), versus utilizing cell-type-specific differential peaks derived from scATAC-seq data (**ATAC**).

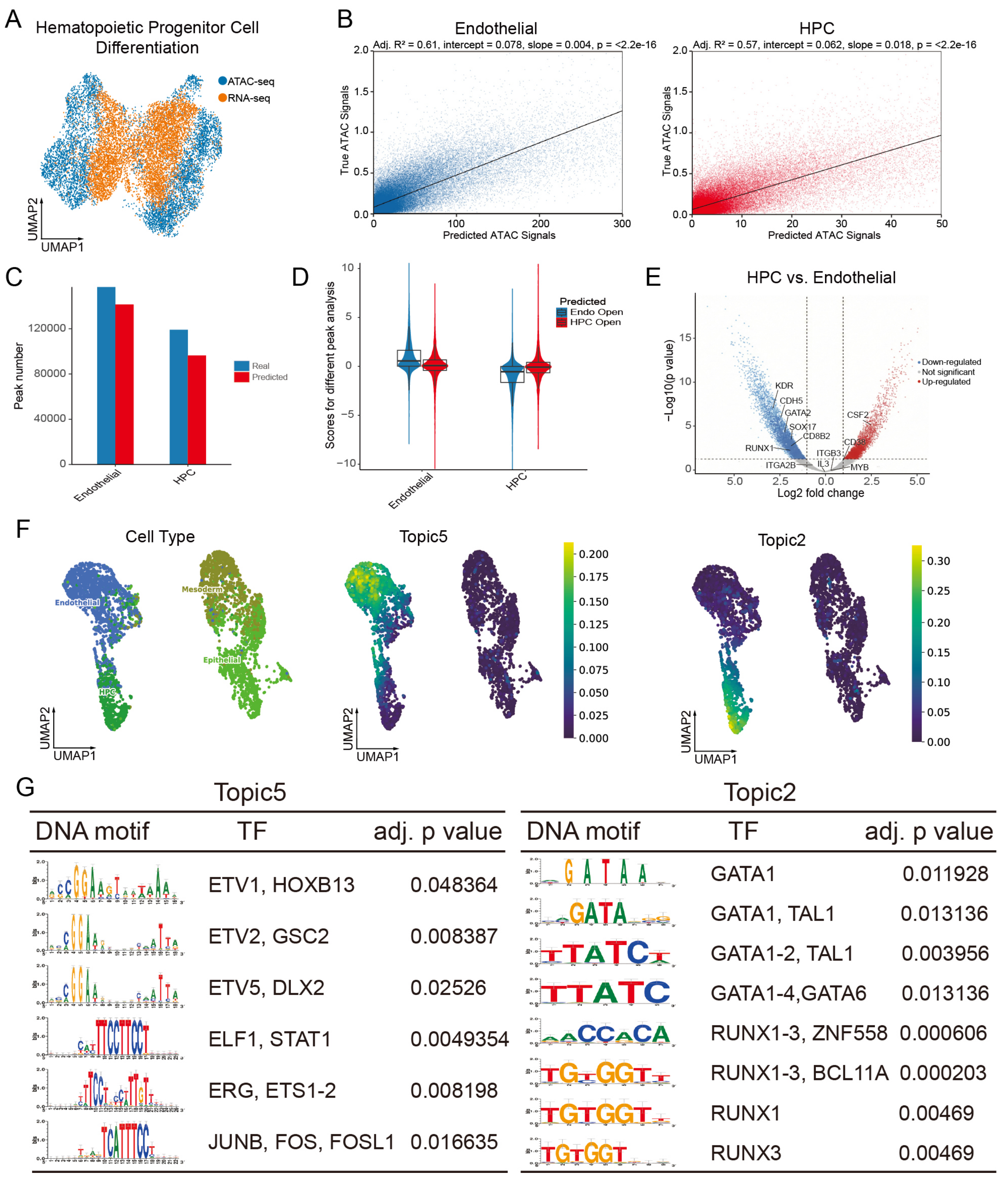

Figure S7 Validation of STED's signal reconstruction accuracy and peak specificity during endothelial-hematopoietic transition. To assess data integration, the endothelial-hematopoietic transition (EHT) dataset is visualized in UMAP space colored by sequencing platform (**A**). The reconstruction accuracy of epiDecon is validated by the correlation between predicted signals and ground-truth scATAC-seq pseudo-bulk profiles for major cell types (**B**), with model fit quantified by the adjusted R-squared ($adj.R^2$). Consistently, the number of peaks predicted by STED for endothelial cells and HPCs aligns closely with those detected in scATAC-seq (**C**). The biological specificity of these predicted peaks is rigorously tested using scATAC-seq data, as illustrated by Wilcoxon rank-sum test scores in violin plots (**D**) and volcano plots contrasting endothelial-specific (Endo) versus HPC-specific signals (**E**). Furthermore, hematopoietic stem cell (HSC) differentiation dynamics are resolved by SCENIC+ UMAPs colored by annotated cell types (left) and pycisTopic assignments (middle and right) (**F**). The functional relevance of these topics is substantiated by tables summarizing significantly enriched transcription factor motifs for Topic 5 (left) and Topic 2 (right) (**G**).

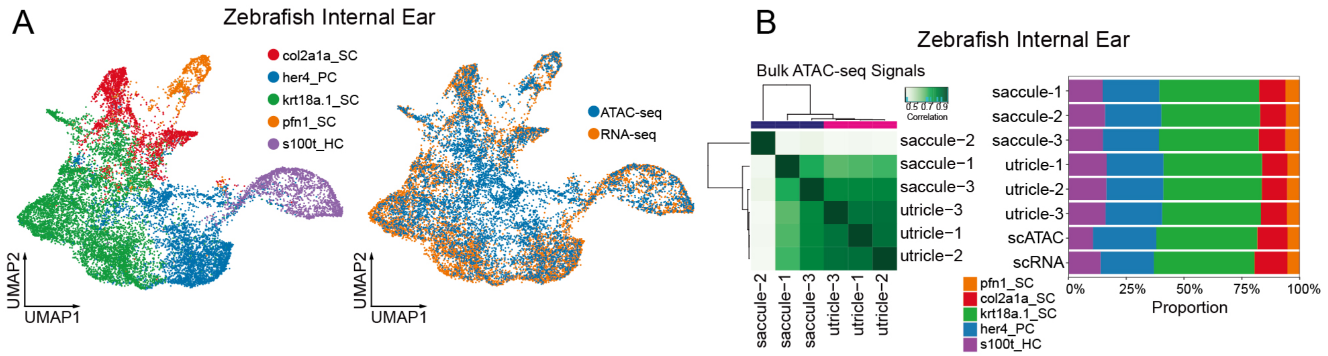

Figure S8 Generalizability of STED in deconvolving the zebrafish inner ear epigenetic landscape. To demonstrate cross-species applicability, the zebrafish inner ear dataset is visualized in UMAP space, revealing distinct clusters colored by annotated cell types (left) and sequencing platform (right) (**A**). The deconvolution performance is rigorously evaluated in this context: consistency between bulk ATAC-seq replicates (utricle and saccule) is visualized via a Pearson correlation heatmap (**B**, left), while the accuracy of cell type abundance inference is validated by comparing epiDecon predictions against single-cell ground truth proportions (**B**, right).

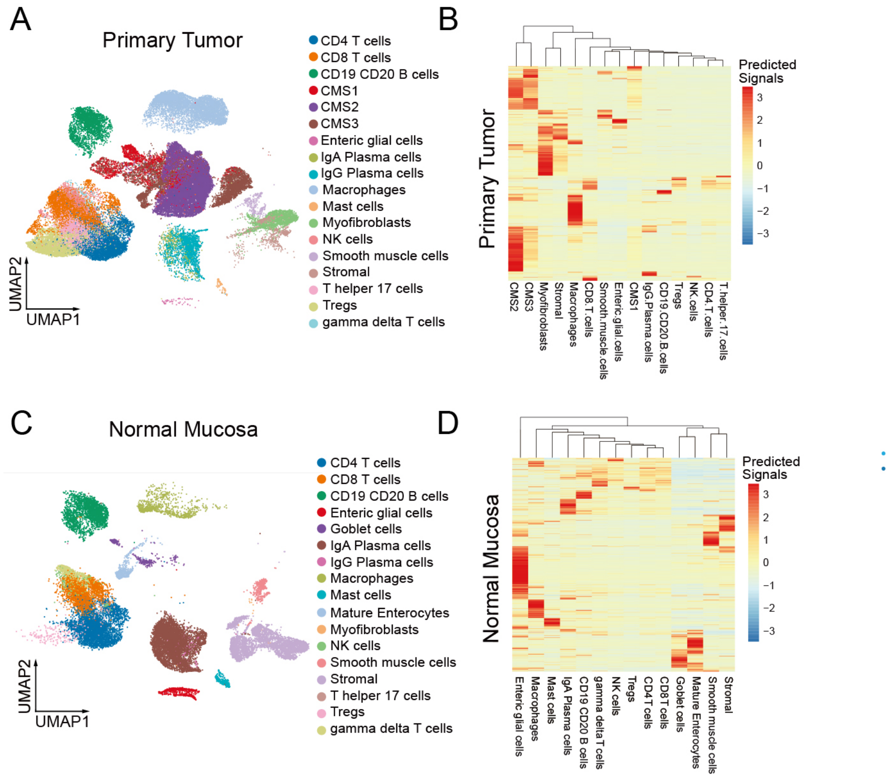

Figure S9 Reconstruction of cell-type specific epigenetic signals in colorectal cancer (CRC). To characterize the epigenetic landscape of CRC, scRNA-seq data from paired tumor and normal tissues are visualized in UMAP space (**A–B**). Leveraging the STED workflow, cell-type-specific H3K27ac signals are inferred and visualized via heatmaps (**C–D**), providing the foundational profiles for downstream regulatory analysis.

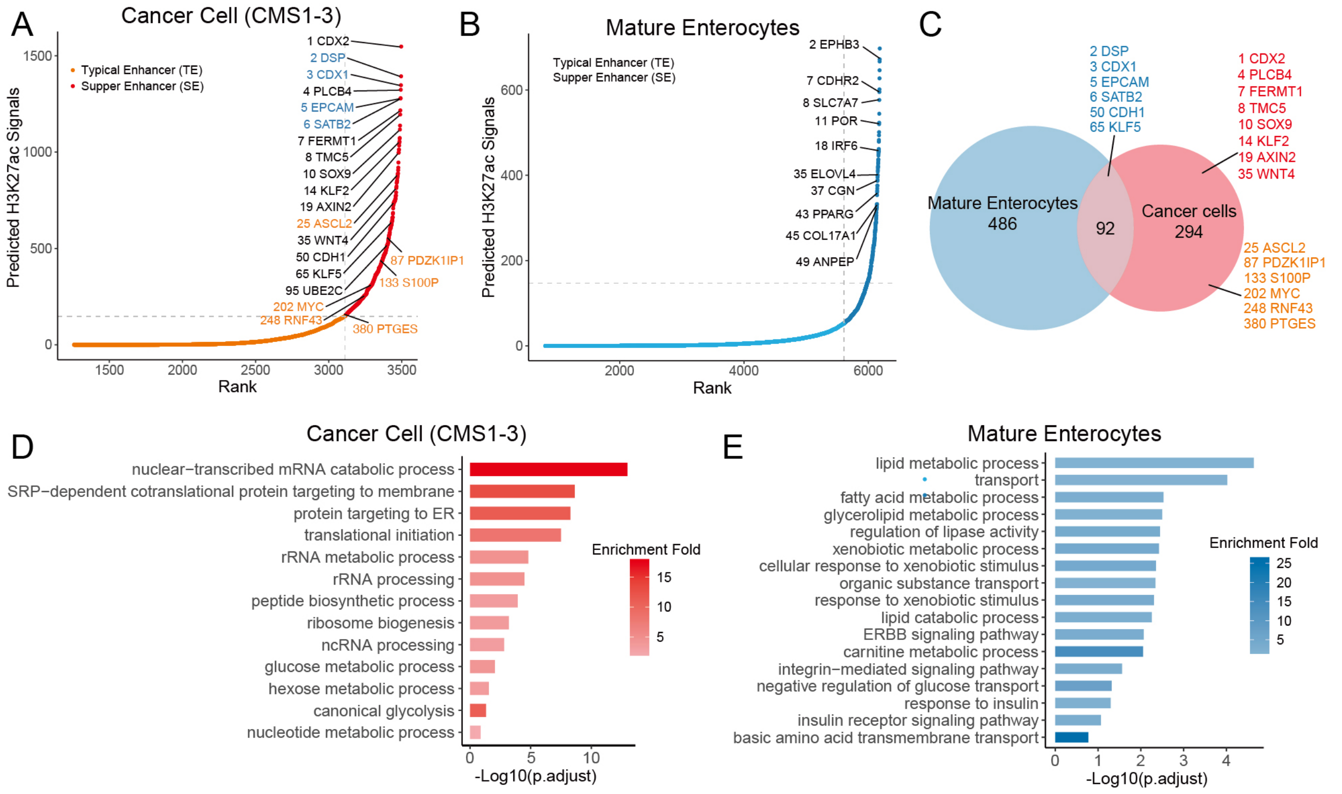

Figure S10 Identification and functional characterization of tumor-driven super-enhancers (SEs). Building on the reconstructed H3K27ac profiles, super-enhancers (SEs) are identified through a ROSE-like rank-ordering approach in tumor cells (**A**) and normal mature enterocytes (**B**). To distinguish disease-specific alterations, a Venn diagram illustrates the overlap of SEs between normal and tumor cells, highlighting shared (Blue), tumor-specific (Red), and validated candidate driver (Orange; Lee et al.) SE-associated genes (**C**). Finally, the biological impact of these regulatory elements is elucidated by functional enrichment analysis using *rGREAT*, revealing the top enriched pathways driven by tumor-specific (**D**) and normal-specific (**E**) SEs.
